# Separation and recovery of exotic radiolanthanides from irradiated tantalum targets for half-life measurements

**DOI:** 10.1371/journal.pone.0235711

**Published:** 2020-07-09

**Authors:** Nadine Mariel Chiera, Zeynep Talip, Adelheid Fankhauser, Dorothea Schumann

**Affiliations:** 1 Laboratory for Radiochemistry, Nuclear Energy and Safety Research Division, Paul Scherrer Institute, Villigen, Switzerland; 2 Analytic Radioactive Materials, Paul Scherrer Institute, Villigen, Switzerland; University of Magdeburg, GERMANY

## Abstract

The current knowledge of the half-lives (*T*_1/2_) of several radiolanthanides is either affected by a high uncertainty or is still awaiting confirmation. The scientific information deriving from this imprecise *T*_1/2_ data has a significant impact on a variety of research fields, e.g., astrophysics, fundamental nuclear sciences, and nuclear energy and safety. The main reason for these shortcomings in the nuclear databases is the limited availability of suitable sample material together with the difficulties in performing accurate activity measurements with low uncertainties. In reaction to the urgent need to improve the current nuclear databases, the long-term project “ERAWAST” (Exotic Radionuclides from Accelerator Waste for Science and Technology) was launched at Paul Scherrer Institute (PSI). In this context, we present a wet radiochemical separation procedure for the extraction and purification of dysprosium (Dy), terbium (Tb), gadolinium (Gd), and samarium (Sm) fractions from highly radioactive tantalum specimens, in order to obtain ^154^Dy, ^157-158^Tb, ^148,150^Gd, and ^146^Sm samples, needed for *T*_1/2_ determination studies. Ion-exchange chromatography was successfully applied for the separation of individual lanthanides. All separations were conducted in aqueous phase. The separation process was monitored *via* γ-spectrometry using suitable radioactive tracers. Both the purity and the quantification of the desired radiolanthanides were assessed by inductively coupled plasma mass spectrometry. Test experiments revealed that, prior to the Dy, Tb, Gd, and Sm separation, the removal of hafnium, lutetium, and barium from the irradiated tantalum material was necessary to minimize the overall dose rate exposure (in the mSv/h range), as well to obtain pure lanthanide fractions. With the herein proposed separation method, exotic ^154^Dy, ^157-158^Tb, ^148,150^Gd, and ^146^Sm radionuclides were obtained in sufficient amounts and purity for the preparation of samples for envisaged half-life measurements. During the separation process, fractions containing holmium, europium, and promethium radionuclides were collected and stored for further use.

## Introduction

Exotic radionuclides with relatively long half-lives are of great interest in a wide range of research domains. Benchmark radionuclides for astrophysics studies are for example the nuclides ^146^Sm, ^148,150^Gd, and ^154^Dy [1–3], whose production (and destruction) pathways provide essential information on the creation and on the development of the known Universe. Long-lived radiolanthanides such as ^157^Tb and ^158^Tb, produced during irradiation of the urania-gadolinia fuel, are of extreme importance in the field of nuclear energy, since the exact knowledge on the nuclear properties of these nuclides is necessary for nuclear fuel disposal and for nuclear transmutation processes [4]. Furthermore, ^157^Tb was lately marked out as a relevant nuclide for the discovery of sterile neutrinos in the keV mass region [5] whereas, in a more futuristic approach, ^158^Tb was suggested as a valid decay-energy source in nuclear accumulators [6]. It is apparent that these studies and applications require precise nuclear data, e.g., the half-lives (*T*_1/2_), of the above-mentioned long-lived radiolanthanides. However, for ^146^Sm, ^148,150^Gd, ^154^Dy, and ^157,158^Tb, only discordant or uncertain information on their *T*_1/2_ is available. As an example, a collection of *T*_1/2_ values for ^146^Sm, ^154^Dy, and ^157^Tb reported in literature is given in Table 1.

**Table 1:** Half-life (*T*_1/2_) values of the exotic radiolanthanides ^146^Sm, ^154^Dy, and ^157^Tb reported in literature.

Nuclide	T_1/2_		Year	Reference
	Value	Uncertainty		
^**146**^**Sm**	~50 My	not stated	1953	[7]
	85 My	12 My	1963	[8]
	74 My	15 My	1964	[9]
	102.6 My	4.8 My	1966	[10]
	103.1 My	4.5 My	1987	[11]
	68 My	7 My	2012	[12]
^**154**^**Dy**	1.5 My	0.9 My	1961	[13] ^a^
	2.9 My	1.5 My	1965	[14]
	10 My	4 My	1967	[15]
	4 My	not stated	1971	[13] ^b^
	3.0 My	1.5 My	1985	[13]
^**157**^**Tb**	160 y	40 y	1963	[16]
	280 y	120 y	1964	[17]
	150 y	30 y	1964	[18]
	99 y	10 y	1983	[19]
	71 y	7 y	1996	[20] ^c^

^a^ revision of [21]

^b^ revision of [22]

^c^ recalculated with data from [19] and [23]

Discrepancies in the available *T*_1/2_ data for ^150^Gd are observed as well, with values differing from one another of at least 20% [10, 24, 25]. For ^158^Tb, former measurements showed estimated uncertainties of 30% [26] and 20% [27], with the currently adopted *T*_1/2_ = 180 ± 11 y [28] still awaiting an independent corroboration. The scientific impact of such uncertain/contradictory values is considerable. A prominent example is ^146^Sm, whose recent *T*_1/2_ re-evaluation might implicate a redetermination of the chronological formation of the Solar System [12]. Currently, a confirmation on this latest measured value is still pending. A similar situation holds for ^148^Gd, for which a more recent *T*_1/2_ = 70.9 ± 1.0 y [29] was estimated. This value, given only as a preliminary result, supports the previous measurement of Prestwood et al. [30]. However, an update on this long-term measurement is still lacking. Reasons for these shortcomings in the nuclear databases are in many cases the limited availability of the isotopes of interest, often accompanied by difficulties in the preparation of samples with a high chemical purity. With the initiative “Exotic Radionuclides from Accelerator Waste for Science and Technology–ERAWAST”, launched by Paul Scherrer Institute (PSI) [31, 32], these limitations are circumvented by using highly active accelerator waste from the PSI accelerator-facilities as a source of exotic radionuclides for scientific purposes. Detailed information on this long-term project can be found at the website “psi.ch/en/lrc/erawast”. PSI owns one of the most powerful accelerators in Europe, i.e., a ring-cyclotron with 590 MeV proton energy and 2.2 mA beam current, which feeds the SINQ neutron spallation source. Between 2000 and 2001, the second SINQ Target Irradiation Program (STIP-II) [33] was performed at PSI, with the purpose of studying radiation damage effects on construction materials induced by high-energy protons and spallation neutrons. During that project, 51 samples of tantalum were exposed to fluxes of high-energy protons and spallation neutrons in the SINQ facility. It is known from previous studies [34–36] that the majority of the radionuclides produced in Ta targets irradiated with protons and spallation neutrons are lanthanides (Lns), with isotopic compositions considerably enriched in neutron-deficient nuclides such as ^146^Sm, ^148,150^Gd, ^154^Dy, and ^157,158^Tb. After a cooling period of almost 20 years, the active Ta material from the STIP-II project represents an extraordinary source of exotic Sm, Gd, Dy, and Tb radionuclides to be used within the scope of ERAWAST. It is important to mention that the method applied for extracting the desired exotic Lns from the radioactive Ta matrix is crucial, since the purity of the retrieved material strongly affects the uncertainty of the half-life determination studies. For the separation of Lns several techniques are known, summarized in [37–39]. In particular, the recovery of Lns from active Ta targets was reported in [40–42]. However, in those studies (mostly involving amounts of Lns in the order of milligrams), the separation efficiency for each Lns was not stated, and furthermore, the applied procedures required an extensive use of organic solvents. Recently, a method for the extraction of ppb amounts of Dy, Gd, and Sm nuclides from proton irradiated Ta targets was reported for cross section measurements [43]. The separated Dy fraction contained traces of Ho and Tb, while the Sm fraction included small amounts of Eu and Pm. However, for the purpose of that work—the determination of production cross sections—a further separation of the lanthanide fractions was not necessary and, hence, was not performed.

Here, we report a chemical separation system for the extraction of Sm, Gd, Tb, and Dy from irradiated Ta samples, in sufficient amounts and purity for future half-life measurements. The separation process was monitored using γ-spectrometry. The purity as well the isotopic composition of each Lns fraction was assessed by inductively coupled plasma mass spectrometry (ICP-MS).

## Experimental

### Materials

#### Irradiated Ta samples

**Fig 1:**
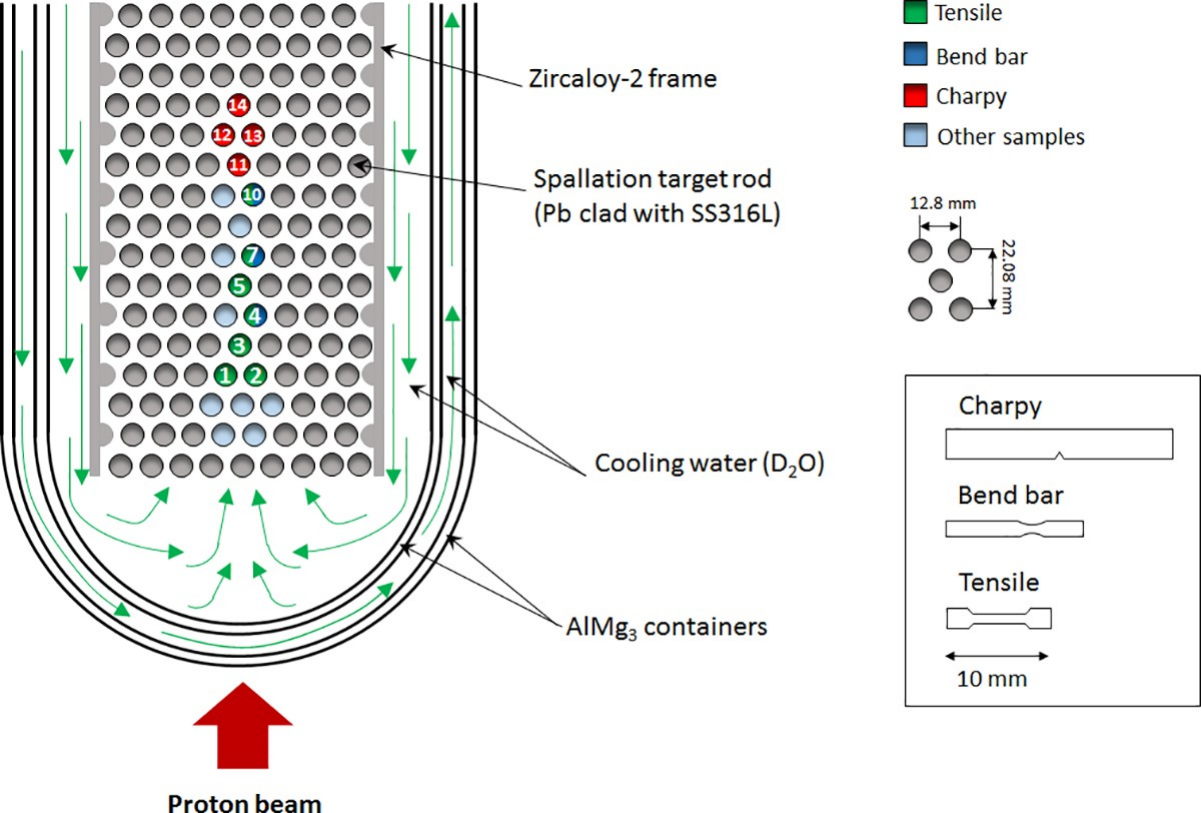
Sketch (not in scale) depicting the irradiation position of each specimen rod in the lower part of the Target-4 of SINQ. The position of the Ta samples classified as “Tensile”, “Bend bar”, and “Charpy” are indicated in green, blue, and red, respectively. The shape of the Ta samples (in scale) is illustrated as well.

Depending on their shapes, the Ta specimens were classified as “Tensile”, “Bend bar”, and “Charpy”. For the irradiation, they were packed in layers in specimen holders, and enclosed in SS 316L tubes. Then, they were inserted in the SINQ target (Target-4), within the Pb spallation target rods, as schematized in Fig 1.

All samples were irradiated for 16 months, with a total proton charge of 10.03 Ah. Further specifications on the irradiation conditions of the Ta specimens (irradiation experiment “IB”) are given in [33]. A list of the STIP-II Ta samples retrieved and available for separation is given in Table 2. In this study, the IB18, IB19, IB22, and IB55 specimens were treated.

**Table 2:** List of Ta samples irradiated during STIP-II and available for separation. The Sample ID refers to the identification number given during the STIP-II studies. The type and the irradiation position of each sample inside the SINQ Target-4 is indicated as well. The samples used in this study are shown in bold.

Sample ID	Type (shape)	Position	Dose rate in contact, mSv/h	Weight per sample, g
IB50	Tensile	3	3	0.13
IB52		3	5	
IB53		3	10	
**IB55**		3	3.53	
IB11	Bend bar	10	20	2.1
IB16		4	40	
IB17		4	80	
**IB18**		10	40	
**IB19**		4	160	
IB20		10	80	
**IB22**		4	80	
IB24		4	40	
IB1	Charpy	12	40	4.3
IB2		12	80	
IB3		12	80	
IB4		12	40	
IB5		14	40	
IB6		14	80	
IB7		14	80	
IB8		14	40	

#### Reagents

Ultrapure water obtained from a Milli-Q2 system water purifier (Millipore Corp., Bedford, MA) was used in all the experiments. Mineral acids were of ACS grade, procured from Sigma-Aldrich (HCl, 37%), Fischer Chemicals AG (HF, 40%), Merck (HNO_3_, 69%), and Alfa Aesar (H_3_BO_3_, 99.99%). Lu standard for ICP, in the form of Lu(NO_3_)_3_, was purchased from Fluka Analytical. 2-hydroxyisobutyric acid (HIBA, Alfa Aesar) solutions in the concentration range 0.09 M– 0.25 M were prepared. All the HIBA solutions were buffered at pH 4.6 by addition of liquor NH_4_ (25%, Merck EMSURE®). The pH of the prepared solutions was measured with a digital pH-meter (Mettler Toledo—InLab® Expert Pro-ISM sensor). During the separation process, the pH of the eluents after passing through the column was determined using pH indicator strips MColorpHastTM (pH 0–14). Cation exchange resins LN (di(2-ethylhexyl)orthophosphoric acid—HDEHP), DGA (N, N, N’, N’-tetrakis-2-ethylhexyldiglycolamide), LN3 (di-(2,4,4-trimethylpentyl) phosphinic acid) extraction resins and Sykam (surface-sulfonated styrene/divinylbenzene copolymers) were used. LN, DGA, and LN3 resins (100–150 μm particle size) were purchased from Triskem International. Macroporous cation exchange Sykam resin (12–22 μm particle size) was supplied from Sykam GmbH. LN, DGA, and LN3 resins were kept in 1 M HNO_3_, while the Sykam resin was conserved in MilliQ grade H_2_O. Before use, the Sykam resin was pre-conditioned with NH_4_NO_3_ (ACS ISO grade, Merck EMSURE®).

#### Radioactive tracers for γ-spectrometry

The separation process was monitored *via* γ-spectrometry. The γ-emitting radionuclides contained in the irradiated Ta targets and their main γ-lines monitored during the separation are reported in Table 3.

**Table 3:** List of γ-emitting radionuclides contained in the irradiated Ta targets. For each radionuclide, the half-life (*T*_1/2_), the energy (E_γ_) and intensity (I_γ_) of the main γ-lines monitored during the separation are indicated. Data taken from [44].

Nuclide	*T*_1/2_	Eγ (keV)	Iγ (%)
^**133**^**Ba**	10.54 y	356	12
^**145**^**Pm**	17.7 y	72.5	1.79
^**150**^**Eu**	36.4 y	333.99	95
^**152**^**Eu**	13.525 y	121.8	28.41
^**158**^**Tb**	180 y	944.2	43.9
^**166m**^**Ho**	1.20E3 y	184.4	74.5
^**172**^**Hf**	1.87 y	125.8	11.3
^**172**^**Lu**	6.70 d	181.53	62.8
^**173**^**Lu**	1.34 y	272	18
^**178m2**^**Hf**	31 y	216.67	64.5

Additionally, γ-emitting Lns tracers were added to monitor the separation of Sm, Gd, and Dy, respectively (see Table 4).

**Table 4:** List of γ-emitting radionuclides tracers added to monitor the separation of Sm, Gd, and Dy. For each radionuclide, the half-life (*T*_1/2_), the energy (E_γ_) and intensity (I_γ_) of the main γ-lines monitored during the separation are indicated. Data taken from [44].

Nuclide	*T*_1/2_	Eγ (keV)	Iγ (%)
^**145**^**Sm**	340 d	61	12
^**153**^**Gd**	240.4 d	97.4	29
^**159**^**Dy**	144.4 d	58	2.29

For each separation, ca 1–5 kBq of each radiotracer was placed in a HDPE scintillation vial, brought to dryness at 70°C under N_2_ flow, and then re-dissolved in 0.5 mL 0.05 M HCl. γ-spectroscopic measurements were performed with a high-purity germanium (HPGe) detector. Energy calibration of the spectrometer was executed with a certified ^152^Eu standard source from PTB (Physikalisch-Technische Bundesanstalt, Braunschweig, Germany). γ-spectra were analyzed with the Genie 2000 Gamma Analysis software.

#### Separation setup

The separation set-up consisted of the solution reservoir (i.e., CELLSTAR® polypropylene tube, capacity 15 ml), a peristaltic pump, a separation column, and the collection vessel (HDPE material, capacity 20 mL). As separation column, single fritted filtration columns (capacity 3 mL, 10–20 μm filter) filled with the resin of interest were used. Bed heights were as follows: LN resin– 3 cm; DGA resin– 1 cm; SYKAM resin– 5 cm; LN3 resin– 1 cm.

### Handling of radioactive material

Separation experiments were performed in the PSI Hotlab, in a lead shielded fume hood, applying the ALARA–As Low As Reasonable Achievable–principle. In each step, the dose rate was kept as 20 uSV/h (measured at the working distance), using dose rate meters 6150AD^®^ Automation und Messtechnik GmbH. The personnel was equipped with personal dosimeters (for the detection of gamma, beta, and neutron irradiation), operational dosimeters (electronic, with immediate dose display and with audible indication of the radiation level when thresholds for dose or dose rates are exceeded), as well extremity dosimeters (finger rings integrating a thermoluminescence-based detector).

### Separation procedure

The first step in the separation procedure concerned the dissolution of the radioactive Ta specimen, followed by the removal of the Ta matrix. Then, two fractional separation methods were applied, namely Method A and Method B. Method A was conceived as a direct fractional separation of the lanthanides after their extraction from the Ta matrix. However, due to the high activity of the treated samples, an alternative separation procedure was considered (Method B). Further details are given in the following sections.

#### Dissolution and removal of the Ta matrix

**Fig 2:**
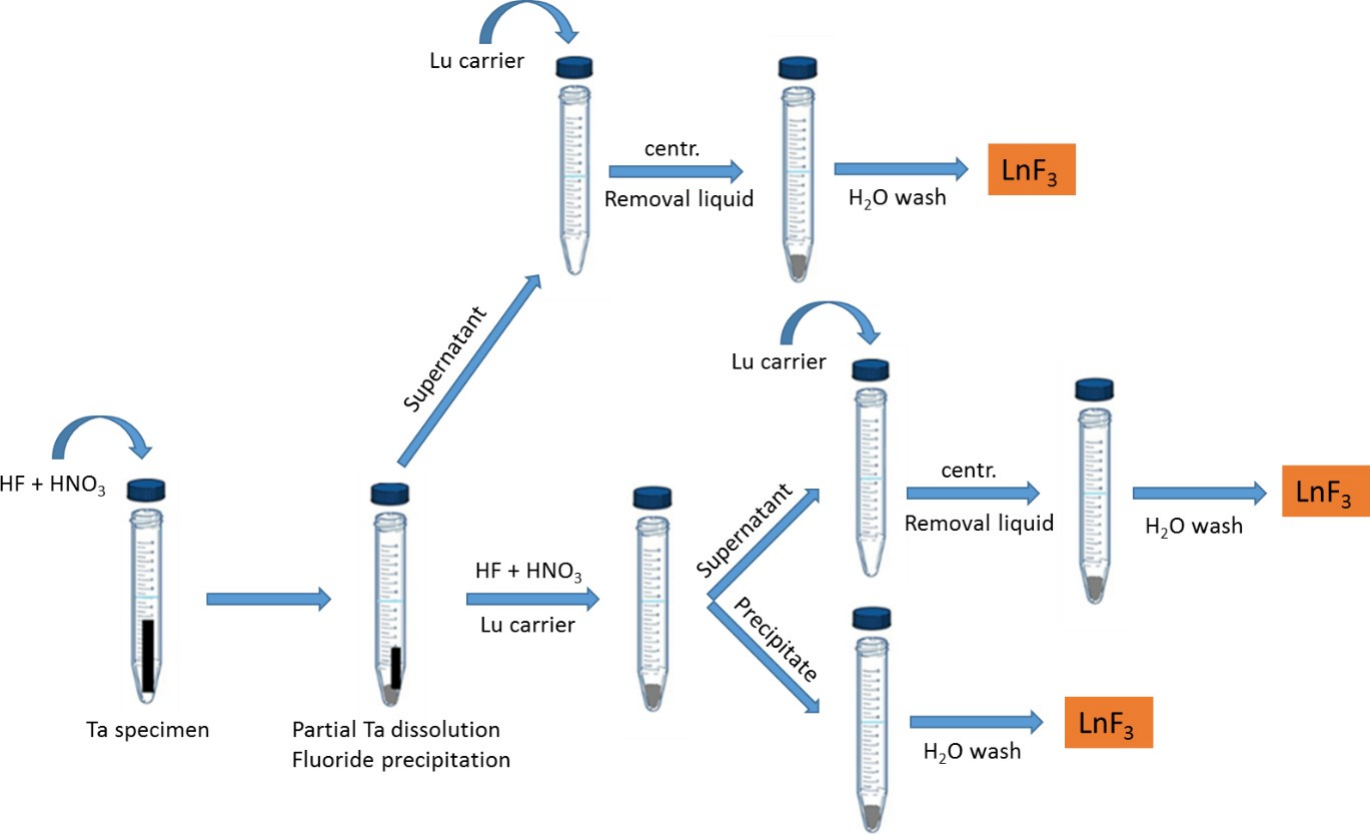
Dissolution scheme of the Ta irradiated samples.

The procedure for the dissolution of the Ta samples and the precipitation of the Lns fluorides is described in detail in [43]. The dissolution of the “Tensile” type specimen was carried out in 4 mL 10 M HNO_3_ and 4 mL conc. HF. When the same mixture of acids was applied to the “Bend bar” type samples, only a partial dissolution together with fluoride precipitation was obtained. The supernatant was thus removed and transferred to another centrifuge tube. Then, the remaining part of the target was dissolved in additional HNO_3_ and HF. To increase the Lns separation yield, precipitation steps were repeated in supernatants by adding Lu carrier. The formation of a LuF_3_ precipitate helped the co-precipitation of the other lanthanides as LnF_3_. The obtained precipitate was separated from the supernatant, rinsed and collected. The precipitate was washed with Milli-Q water to remove any remaining Ta. The scheme for the LnF_3_ precipitation is shown in Fig 2.

LnF_3_ was dissolved in a centrifuge tube with 1 mL 0.5 M H_3_BO_3_ and 1 mL 6 M HNO_3_. H_3_BO_3_ was used to bind any remaining HF and to facilitate the dissolution of the aqueous insoluble lanthanide fluorides by forming tetrafluoroboric acid in solution [45]. To facilitate the dissolution the tube was placed in a water bath at 80°C. After dissolution, alternatively Method A and Method B were applied in order to sequentially isolate the desired Lns fractions from the LnF_3_ precipitate.

#### Separation Method A

**Fig 3:**
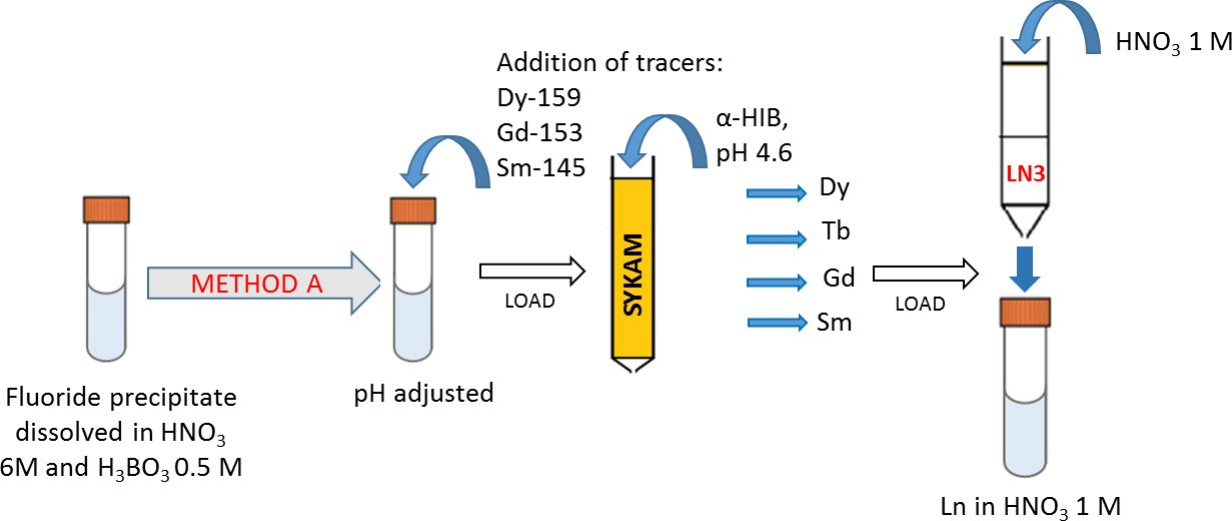
Separation Method A. The separation of the lanthanides of interest from the fluoride precipitate is performed with a two-step separation procedure (SYKAM+LN3 resins).

A schematic representation of the separation Method A is given in Fig 3.

The pH of the dissolved LnF_3_ was adjusted to 1 with 3 M NH_4_OH, and the γ-emitting tracers were added. The solution was then loaded into the Sykam macroporous cation exchange resin. After loading, ~12 mL of 1 M NH_4_NO_3_ was used to bring the column pH to ~5. Fractional Lns separation was performed with a gradient elution technique using different HIBA concentrations. Elutions were performed at a flow rate of 0.4 mL/min. Each eluted fraction, containing 0.8 mL, was measured by γ-spectrometry. After completion of the Lns separation, the fractions containing the same element were combined. To remove the complexing agent (HIBA) from each Lns fraction, LN3 extraction resin was used as a last step. The purified lanthanides fractions were separately eluted in 1 M HNO_3_. The purity of the so-obtained Sm, Gd, Tb, and Dy fractions was then evaluated *via* ICP-MS.

#### Separation Method B

**Fig 4:**
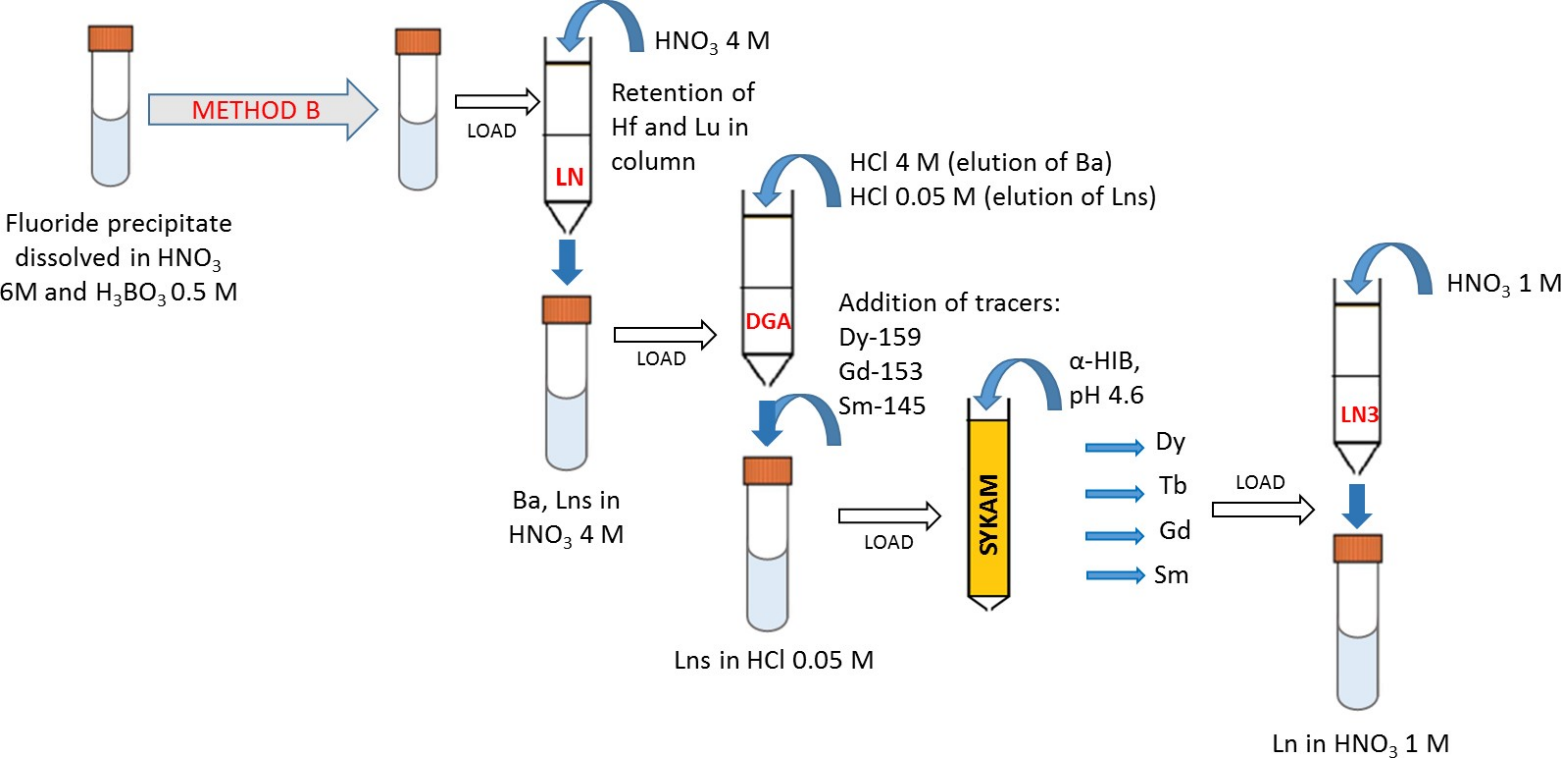
Separation Method B. The separation of the lanthanides of interest from the fluoride precipitate is performed with a four-step separation procedure (LN+DGA+SYKAM+LN3 resins).

A schematic representation of the separation Method B is given in Fig 4.

The dissolved LnF_3_ was loaded into the LN resin. Hf and Lu were retained in the column, while Ba and the Lns lighter than Lu were eluted with 4 M HNO_3_. The fractions containing Ba and the Lns were combined for subsequent separation. Then, Lu and Hf were eluted with 6 M HNO_3_ and 10 M HNO_3_, respectively. The solution containing Ba and the Lns was loaded into DGA resin. The Lns were retained in the column, while the eluted solution (4 M HNO_3_) contained only Ba. After changing the eluent to chloric acid, further elution with 4 M HCl allowed for the complete removal of Ba from the DGA resin. The Lns were collected using 0.05 M HCl as eluent. This solution was successively loaded into the Sykam ion exchanger resin. No pH adjustment of the loading solution was performed. The pH of the column was adjusted to 5 by passage of ca 30 mL of 0.09 M HIBA. Lns were fractionally eluted from the Sykam resin with HIBA *via* a gradient elution technique, as in Method A. Each Lns fraction was then transferred to nitrate form by using LN3 resin and 1 M HNO3 as eluant. The purity of the so-obtained Sm, Gd, Tb, and Dy fractions was analyzed by ICP-MS.

### ICP-MS measurements

The ICP-MS measurements were performed using an Element II® Sector-Field Inductively Coupled Plasma Mass Spectrometer (SF-ICP-MS) by Thermo Fisher. The samples were diluted in 0.28M HNO_3_ prepared from high purity nitric acid and Milli-Q water. Dilution factors (DF) of 10, 50, or 100 were chosen after pre-measurements (see Table 5).

**Table 5:** Dilution factors (DF) for the ICP-MS analysis of the Dy, Tb, Gd, and Sm fractions obtained with Method A and Method B.

Fraction	DF, Method A	DF, Method B
**Dy**	50	100
**Tb**	10	100
**Gd**	10	100
**Sm**	13	100

This step was necessary for having in the measurement solution a suitable amount of the main isotopes to determine their concentration, while still being able to detect other lanthanide impurities by scanning the background. As an internal standard, a certified standard solution with 10 mg/L Re was added (ESI, Elemental Scientific, Omaha, NE, USA). The concentrations of the main isotopes in the solution were determined with an external multi-element standard (TraceCERT®, Periodic Table Mix 1, 2, and 3) prepared in different concentrations (dilution series). The ICP-MS settings (gas flows, torch position, and lenses) are tuned daily by introducing an in-house multi-element solution. From this solution, B, Rh, and U (1 ppb each) are used to optimize intensity, peak shape, and U/UO value to minimize oxide formation (3–4%). The mass offset for each isotope was checked by measuring the multi-element standard solution after tuning the machine. These mass offsets in the range of -0.1 to +0.1 were imported to the operation method. The operation method was set to scan the masses between 116 and 187 several times in low- and medium-resolution. Instrument settings and data acquisition parameters are shown in Table 6.

**Table 6:** Operating conditions and data acquisition parameters used for the ICP-MS measurements.

**Inlet system**	ESI SC-μ Autosampler with PTFE tubing
	MicroFlow PFA-ST nebulizer, uptake rate ~70μl/min
	ESI PFA Conical Spray Chamber
	2mm-Sapphire Injector tube with Quartz torch
	Nickel sampler and skimmer cones
**Forward RF power**	1350 W; Guard electrode on
**Typical Argon gas flows**	Cool gas: 16.5 L/min
	Sample gas: 0.800–0.810 L/min
	Auxiliary gas: 0.900 L/min
	Additional gas: 0.200–0.220 L/min
**Resolution**	Low (LR) = 300 m/Δm
	Medium (MR) = 4000 m/Δm
**Data acquisition**	Mass window: 50% (LR); 60% (MR)
	60% at Medium Resolution
	Search/Integration window: 80%/20%
	Integration type: average
	Samples per peak: 20–30
	Settling time: 0.001–0.3 s
**Detection mode**	Analog and ion counting on secondary electron multiplier (SEM)
**Masses measured**	LR: 116 to 124; 134 to 170; 172 to 181; 185 and 187
	MR: 134 to 170; 175; 177 to 181; 185 and 187
**Number of scans**	17 scans
**Measuring time per sample**	6:22 minutes
**Wash time between samples**	10 minutes with 3% HNO_3_

In the measurement sequence, the samples are bracketed with the measurement of the multi-element standard dilution series. Each sample, as well each standard series, is preceded by a HNO_3_-blank. Each sample was measured twice independently. The detection limit of each isotope is calculated with three times the standard deviation of the Blank measurements and the dilution factor of the samples is considered. The results of the measurement are given as intensities for each mass in counts per second (cps), average of the scans. All count rates are corrected for machine background contributions by subtracting the signals previously measured on pure acid solutions. Internal normalization to cancel drift in responsiveness of the machine is done relative to ^185^Re (internal standard). Then, the normalized count rate *versus* the concentration for each mass of the external multi-element standards series (measured before and after the samples) is interpolated and applied to calculate the concentration (ppb) of each sample. The relative uncertainty of the concentrations is empirically stated as 10% (2σ). The latter includes the variations of standard measurements treated as samples, the weighing uncertainties in the preparation of the standard and sample solutions, as well the uncertainty of the concentrations in the external standard (≤ 0.8%, 2σ).

## Results and discussion

**Fig 5:**
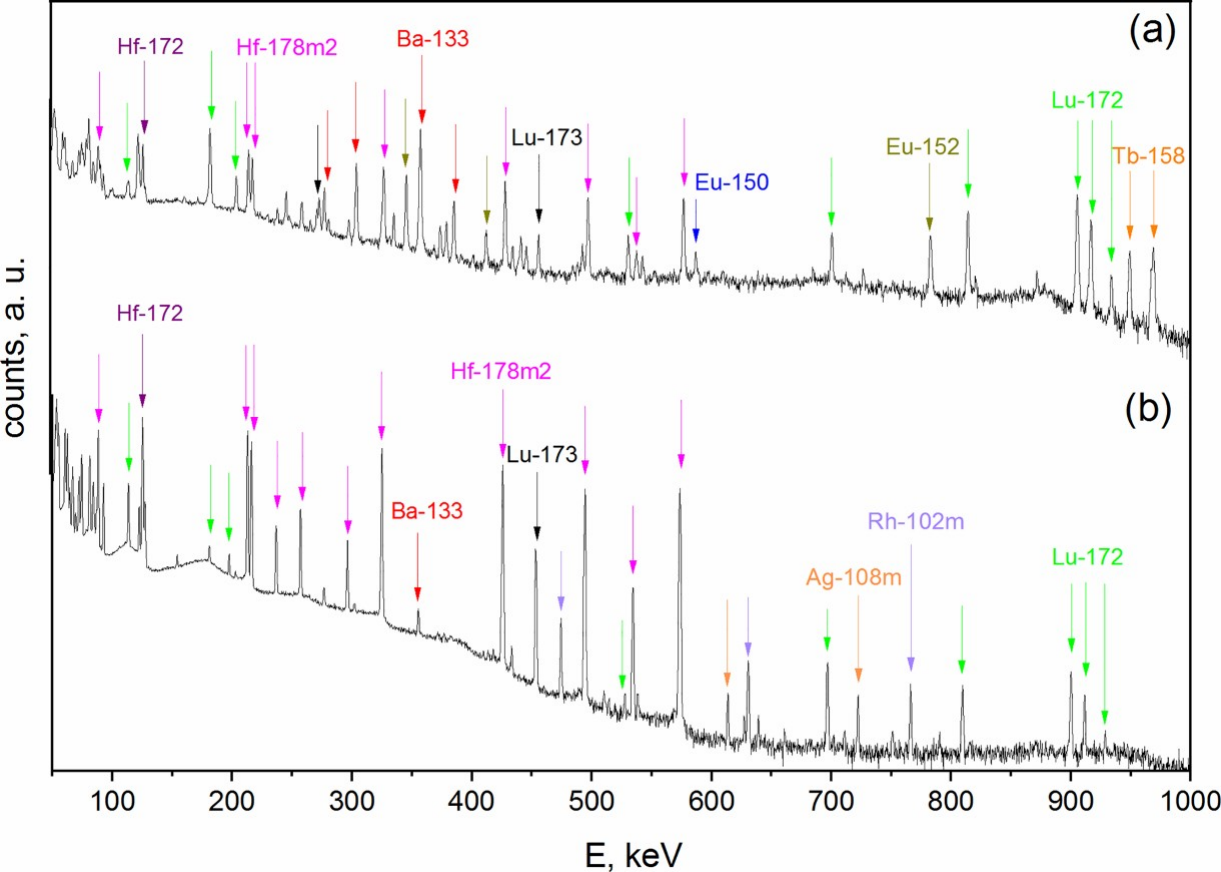
Exemplificative γ-spectrum of (a) the LnF_3_ precipitate, and (b) the supernatant after fluoride precipitation.

Due to the long time elapsed after irradiation of the Ta samples (i.e., 17 years) short-lived reaction products were already decayed. Exemplificative γ-spectra of the LnF_3_ precipitate and the supernatant after fluoride precipitation are shown in Fig 5.

As in [43], an average 98% Lns separation yield from the Ta matrix was here obtained. The detection of ^133^Ba and ^178m2^Hf radionuclides in the LnF_3_ precipitate (Fig 5A) is due to the sorption of Hf and Ba cations on the negatively charged surface of the LuF_3_ carrier, that ultimately leads to the co-precipitation of Hf and Ba in the form of fluoride complexes [46]. The LnF_3_ precipitate showed a contact dose rate between 0.5 and 30 mSv/h, depending on the type of Ta target initially dissolved. This dose-rate mainly derived from the decay of ^172^Hf (and its daughter ^172^Lu), ^178m2^Hf, ^173^Lu, and ^133^Ba.

**Fig 6:**
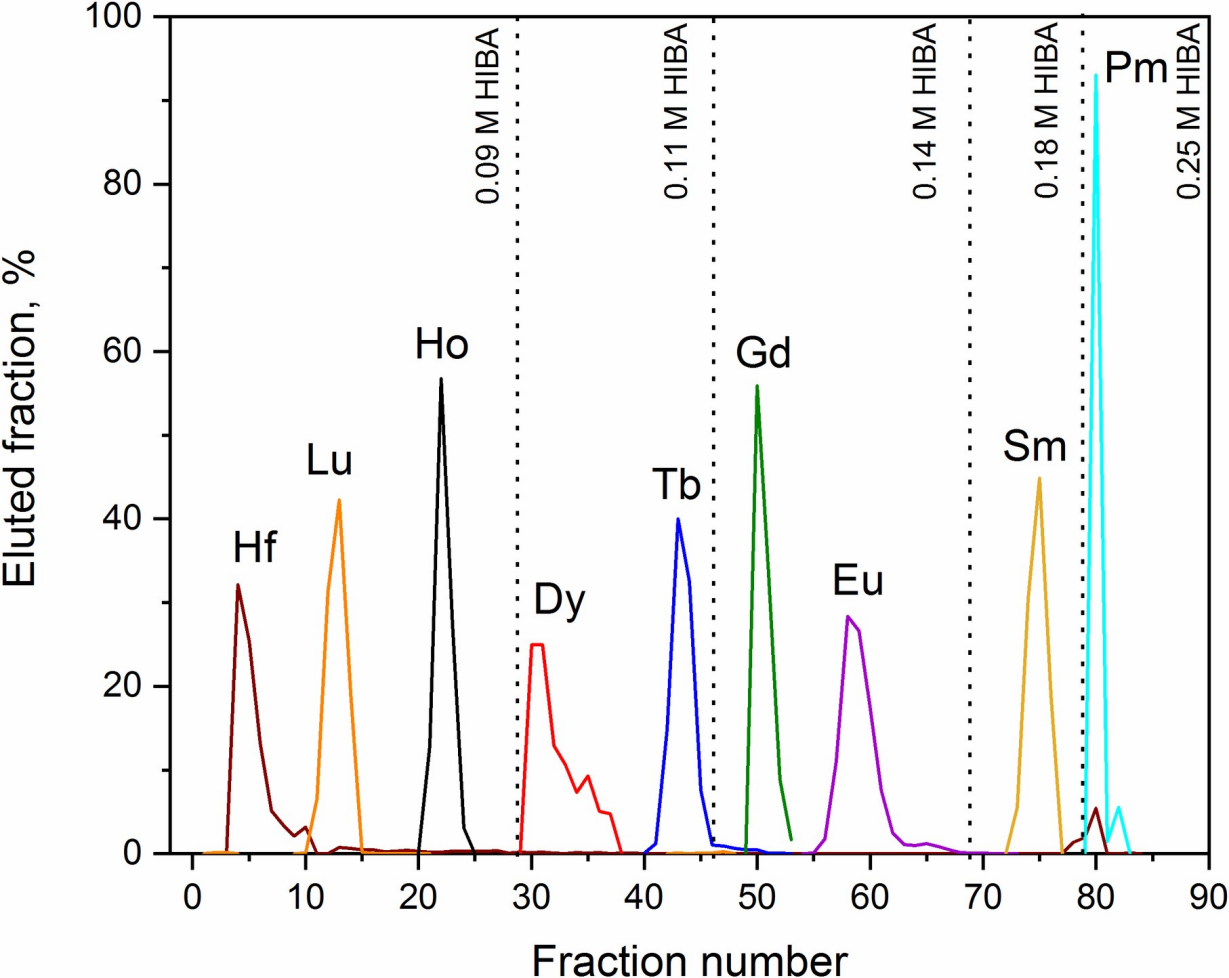
Fractional separation of Ho, Dy, Tb, Gd, Eu, Sm, and Pm fractions with Method A. Separation of the Lns was performed *via* a gradient elution with HIBA at pH = 4.6 on SYKAM resin.

In Method A, the dissolved LnF_3_ was directly loaded into the Sykam resin. Prior to the loading, the pH adjustment of the sample was performed since the distribution coefficients (K_d_) of Lns in cation-exchange resins increase with lowering the acidity of the loading solution. At such conditions, the competition for the resin binding sites between the lanthanide ions and the hydroniums of the loading solution is minimized, and thus, all the Lns are retained in the resin phase. After passage of 1 M NH_4_NO_3_ and ~10 mL 0.09 M HIBA, the pH of the column was adjusted to ~5. Only at this point, the elution of the Lns was observed. This is due to the fact that at strong acidic conditions the -COOH functional group of HIBA exists predominantly in un-dissociated form, and thus the ligand shows a poor coordination strength [47]. The extraction of the Lns from the resin was performed by complexation with HIBA. An example of the separation profile with Sykam resin is presented in Fig 6.

The removal of the Lns from the Sykam resin was performed by applying a gradient elution technique. With increasing the HIBA concentration, the number of molecules in the mobile phase capable of chelating the Lns (III) ions is augmented. This results in a lower affinity of the Lns ions for the cation exchanger, and hence, in their elution from the separation column. Because of the lack of Nd, Pr, Ce, and La γ-emitter tracers, these elements could not be individually separated and hence were eluted together with Pm by using 0.25 M HIBA, and collected in the “Pm fraction”. Satisfactory peak separations were obtained at an elution flow rate of 0.4 mL/min. At higher flow rates, overlap of peaks due to longitudinal diffusion were observed. After performing the fractional separation, each lanthanide of interest was eluted from the Sykam resin within _~_20 mL of HIBA. After the Lns separation, Ba was stripped from the column with 4 M HNO_3_. The concentration of each lanthanide in a reduced volume (_~_5 mL) of 1 M HNO_3_ was performed with the LN3 resin column. The upload in HIBA phase exploited the high affinity of the resin towards Lns(III) ions in low concentrated acids [48], whereas 1 M HNO_3_ was sufficient to elute the Lns.

**Fig 7:**
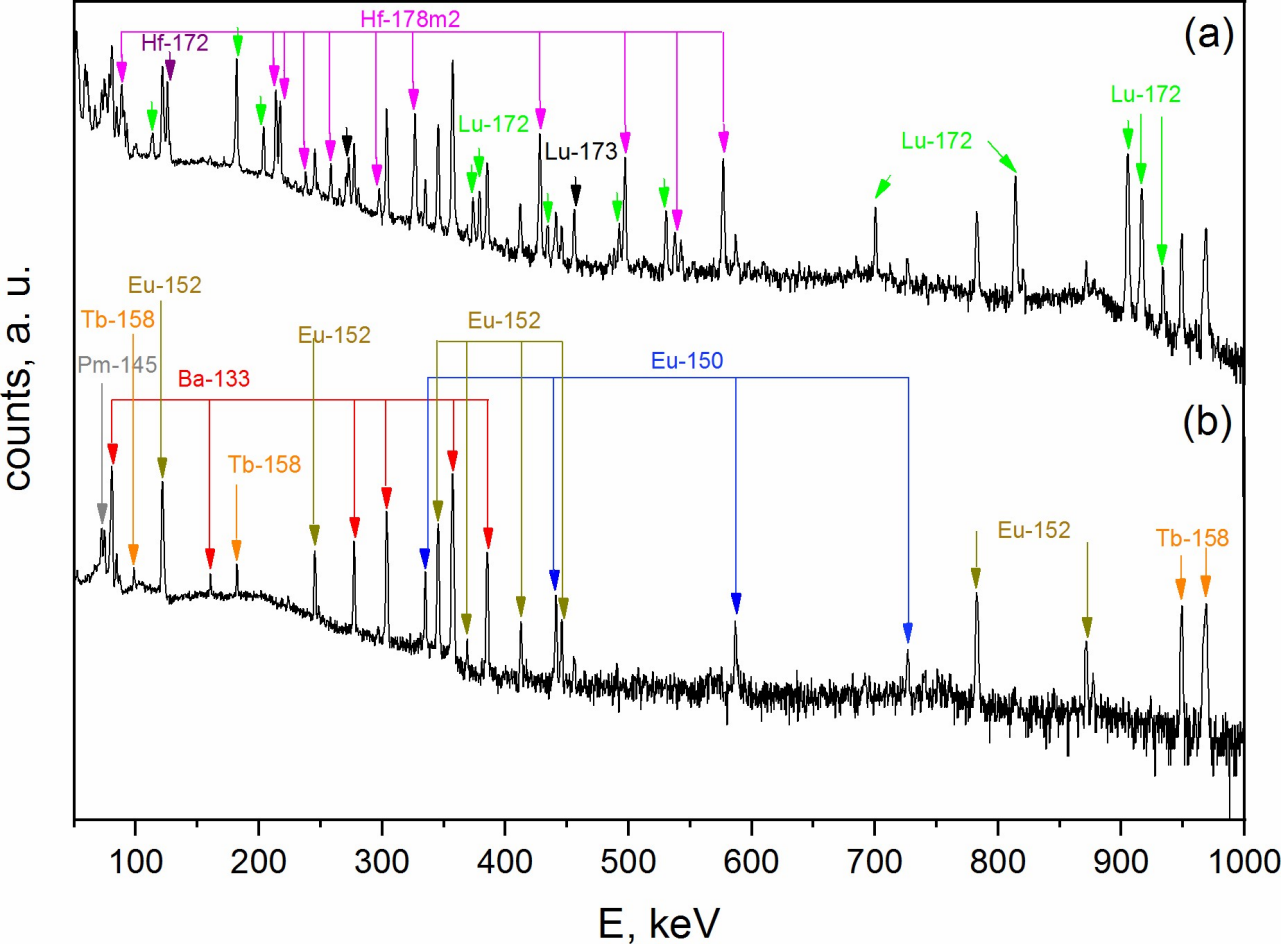
Exemplificative γ-spectrum (a) before and (b) after the separation of Hf and Lu from the LnF_3_ precipitate.

In the separation Method B, the extraction of Hf, Lu, and Ba from the fluoride precipitate was prioritized in order to decrease the dose rate to which the personnel was exposed. For the removal of Hf and Lu, LN resin was chosen due to the high K_d_ of Hf(IV) and Lu(III) on HDEHP resin [49, 50]. An example of γ-spectra showing the qualitative separation of Hf and Lu with LN resin is shown in Fig 7.

**Fig 8:**
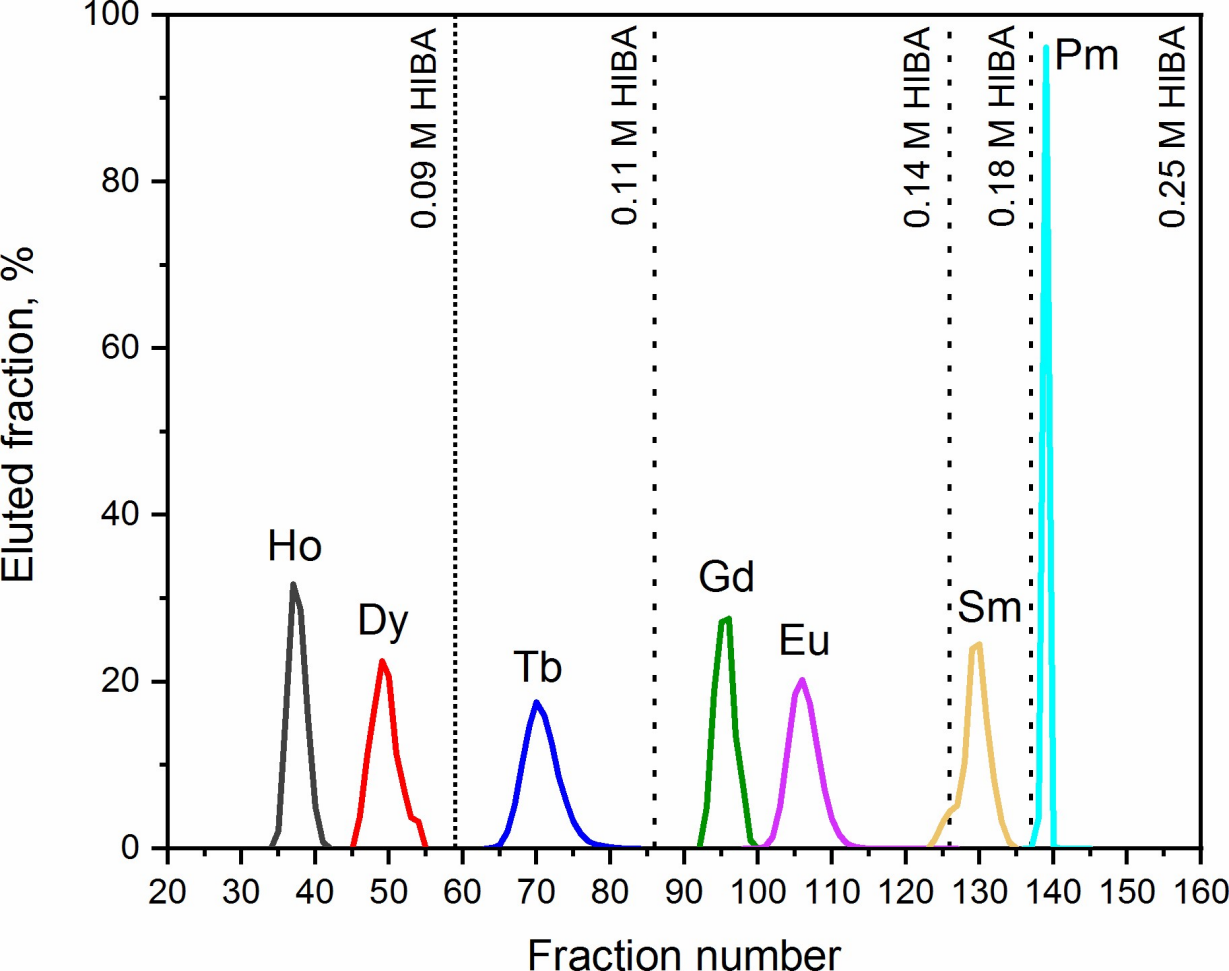
Fractional separation of Ho, Dy, Tb, Gd, Eu, Sm, and Pm fractions with Method B. Separation of the Lns was performed *via* a gradient elution with HIBA at pH = 4.6 on SYKAM resin.

With this separation step, a reduction of ~80% of the initial emitted dose was achieved. After elution from the LN resin, the Ba and the Lns fraction (4 M HNO_3_) was directly loaded onto the DGA resin. It is known that Lns(III) show a high affinity for the DGA resin, regardless of the used HNO_3_ concentration [51]. At the same conditions, Ba(II) is instead not retained. In order to elute the Lns from the resin, a different mobile phase is thus needed. It was previously shown that Lns heavier than Nd have a log_10_(K_d_) > 3 on DGA resin for HCl concentrations above 4 M [51]. However, when the concentration of HCl is lowered, the affinity of Lns towards the DGA resin is dramatically reduced. Thus, the elution of the Lns was pursued with 0.05 M HCl. The collected Lns were directly loaded to the Sykam column, previous addition of the γ-tracers. Due to the low acidity of the 0.05 M HCl solution, its pH adjustment was not necessary. After loading the Lns in the Sykam column, passage of ~35–40 mL of 0.09 M HIBA was in average required to adjust the pH of the column to _~_5. The Lns fractional separation followed the same procedure as previously described in Method A, with the difference that no Hf and Lu elution peaks were observed (Fig 8).

**Fig 9:**
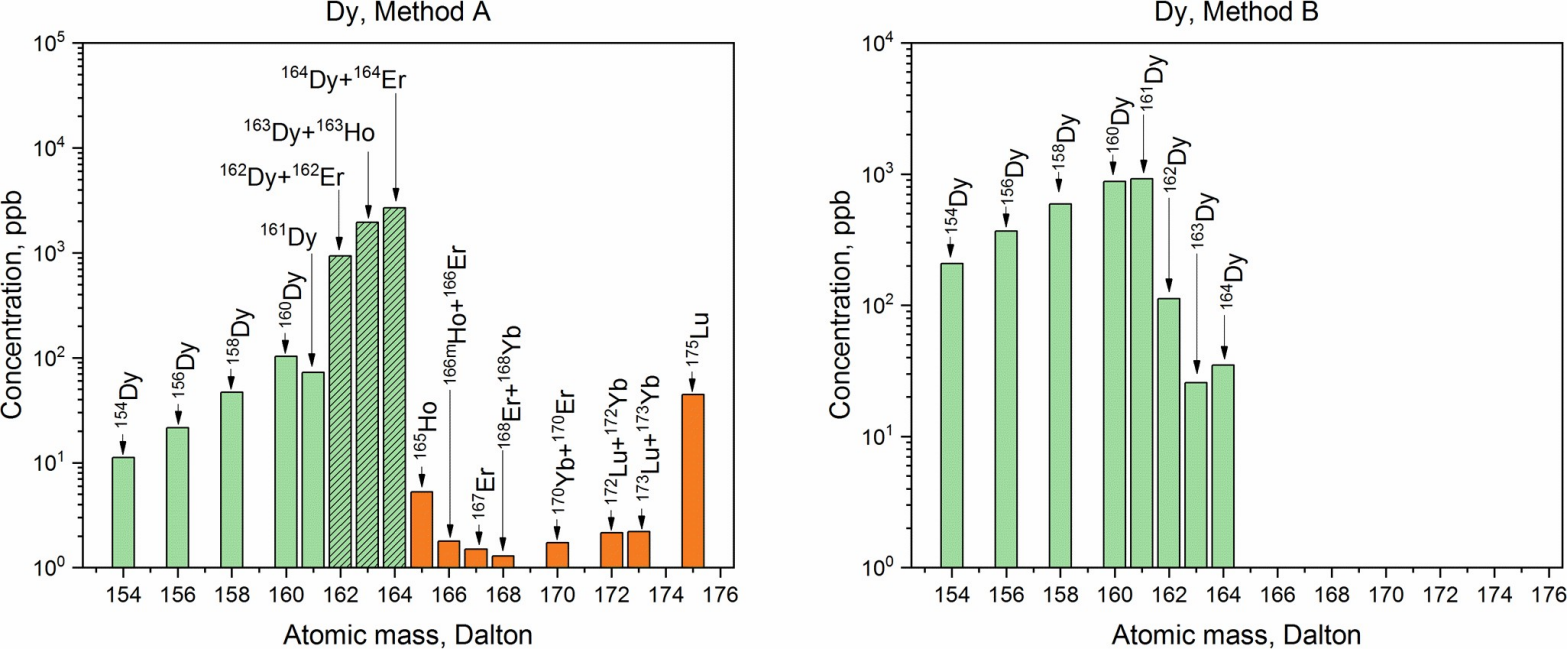
ICP-MS spectra of the dysprosium fractions obtained with Method A and with Method B. Dy isotopes (green bars), other lanthanides and hafnium (orange bars), isobaric interferences for Dy (green patterned bars).

**Fig 10:**
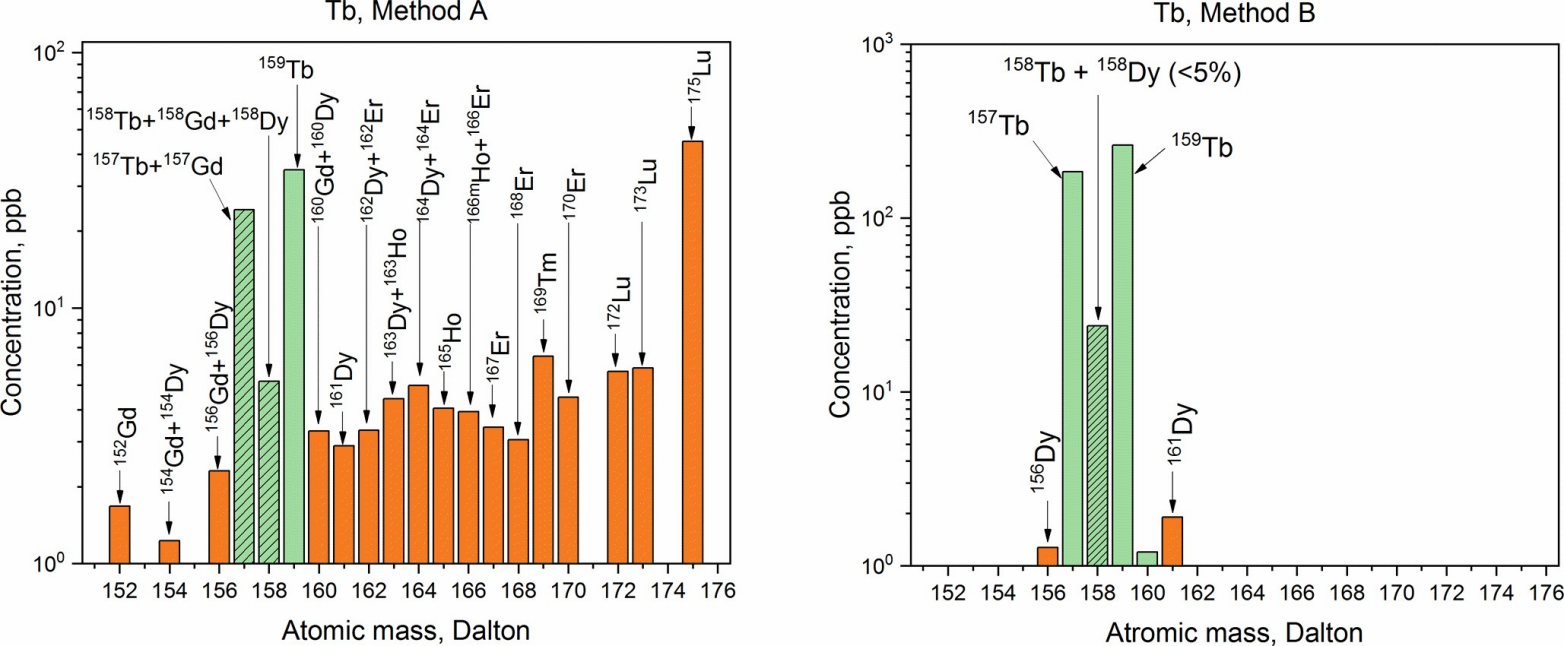
ICP-MS spectra of the terbium fractions obtained with Method A and with Method B. Tb isotopes (green bars), other lanthanides (orange bars), isobaric interferences for Tb (green patterned bars).

**Fig 11:**
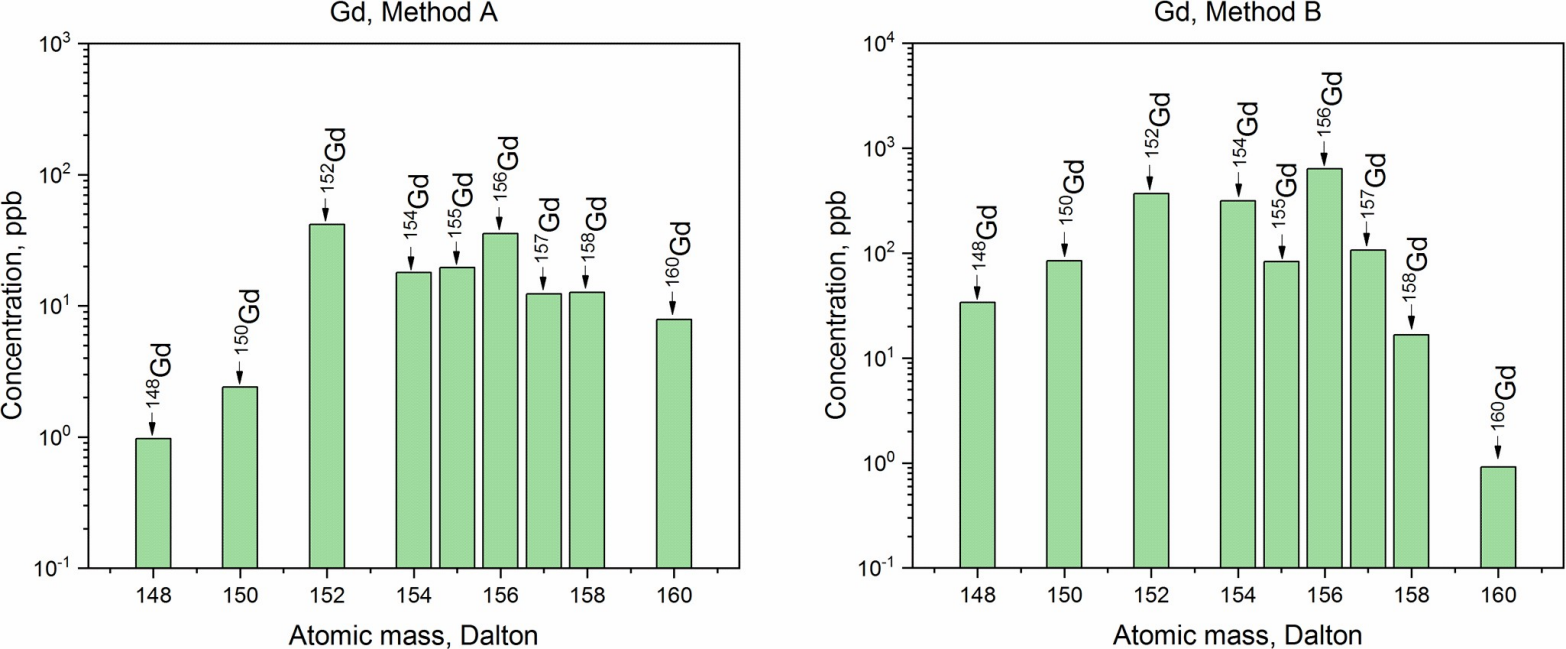
ICP-MS spectra of the gadolinium fractions obtained with Method A and with Method B.

**Fig 12:**
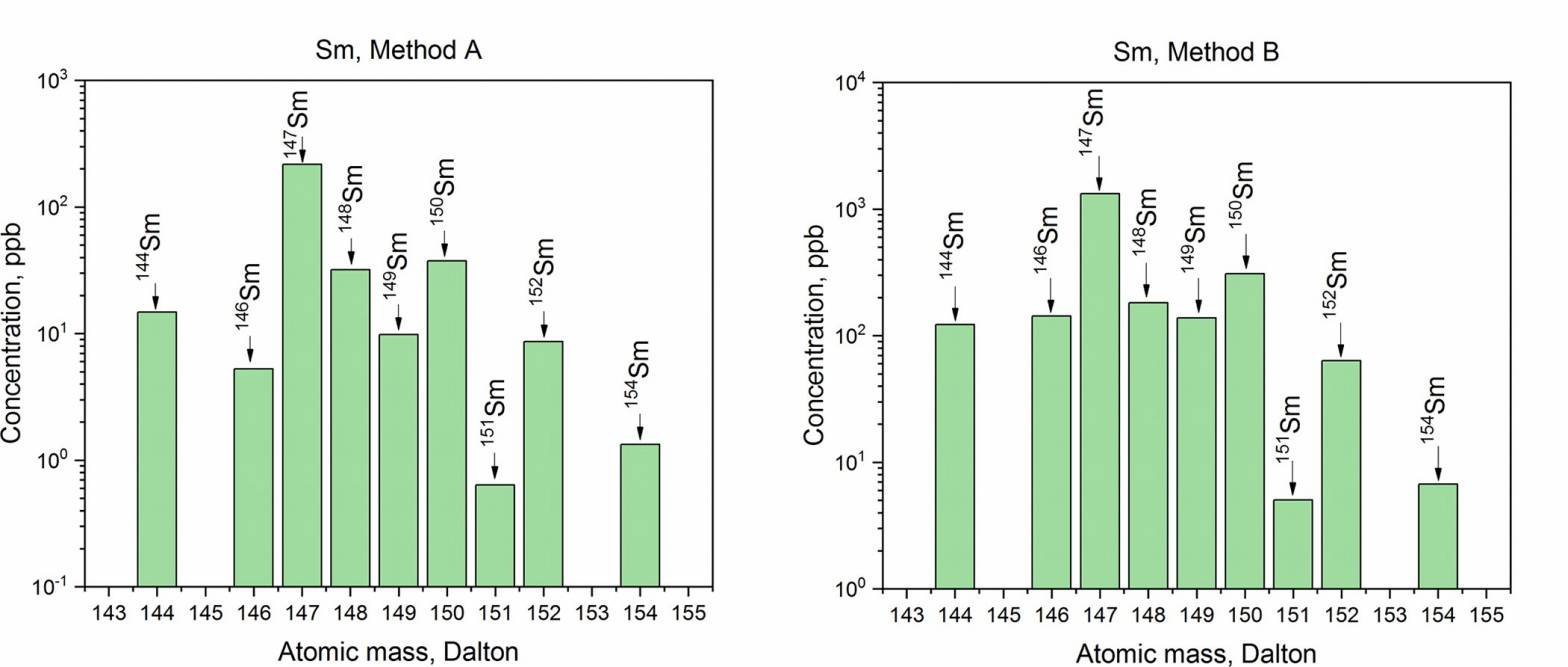
ICP-MS spectra of the samarium fractions obtained with Method A and with Method B.

The purity of the collected Dy, Tb, Gd, and Sm fractions was analyzed by ICP-MS. Comparative ICP-MS results are shown in Figs 9–12. Only signals above the limit of detection (LOD) were considered.

Chemical purity of each fraction was checked by assessing the absence/presence of the nuclides reported in Table 7.

**Table 7:** Masses of reference (in Dalton), corresponding to nuclides not affected by isobaric interference. The identification of the reported mass signals in the ICP-MS spectra corresponds to the univocal presence of a specific element and thus, to the related stable and long-lived isotopes.

Mass(es) of reference (Dalton)	Assigned nuclide(s)	Related isotopes
142 and 143	^142,143^Nd	^144,145,146,148,150^Nd
151 and 153	^151,153^Eu	^150,152,154,155^Eu
159	^159^Tb	^157,158^Tb
165	^165^Ho	^163^Ho, ^166m^Ho
169	^169^Tm	No other stable or long-lived (t_1/2_>1y) isotope
175	^175^Lu	^173,174,176^Lu +^172^Lu (as ^172^Hf daughter)
147 and 149	^147,149^Sm	^144,146,148,150,151,152,154^Sm
155 and 156	^155,156^Gd	^148,150,152,154,157,158,160^Gd
161	^161^Dy	^154,156,158,160,162,163,164^Dy
167	^167^Er	^162,164,166,168,170^Er
171	^171^Yb	^168,170,172,173,174,176^Yb
178	^178^Hf	^174,176, 177, 178m2, 179, 180^Hf

These nuclides were selected as benchmarks since they are not affected by isobaric interference, i.e., each atomic mass (A) corresponds only to a single nuclide.

In all the fractions, the disruption of the natural isotopic abundance of each element, with a predominance of neutron-deficient isotopes, was observed. ICP-MS analysis showed a significant presence of Lu (_~_100 times the LOD) in the Dy and Tb fractions obtained with Method A. The high amounts of Lu contained in the LnF_3_ precipitate led to the tailing of the Lu elution peak, which affected the quality of the Dy and Tb fractions. Presence of Hf nuclides (signals at A = 178 Dalton and A = 179 Dalton) in the separated Dy was recorded as well. In the same fraction, the signal at A = 165 Dalton related to Ho was detected and, as a consequence, the presence of the long-lived isotope ^163^Ho (*T*_1/2_ = 4570 y [44]) cannot be excluded. Thus, isobaric interference ^163^Dy-^163^Ho at A = 163 Dalton is expected. Signal at A = 167 Dalton (4 times the LOD) was recorded as well. This indicates Er impurities in the Dy fraction. Hence, isobaric interferences ^162^Dy-^162^Er and ^164^Dy-^164^Er are presumed. In the Tb fraction, the presence of Gd (concomitant detection of signals at A = 155 Dalton and A = 156 Dalton) and Dy (signal at A = 161 Dalton) was found. It follows that the isobaric interferences ^157^Tb-^157^Gd at A = 157 Dalton and ^158^Tb-^158^Gd-^158^Dy at A = 158 Dalton are expected. By increasing the Sykam column bed height, a better separation resolution might be achieved when an initial Hf, Lu, and Ba separation from the LnF_3_ precipitate is not applied. However, this would comport a considerable increase of the time required for the separation, and hence, an unnecessary exposure of the personnel to high dose rates.

In the Sm, Gd, and Dy fractions obtained with Method B, no isobaric interferences are detected. In the Tb fraction, however, a signal 3 times above the LOD at A = 161 Dalton–i.e., ^161^Dy–was recorded. Due to the detection of Dy in the sample, the presence of the ^158^Dy isotope cannot be excluded. From the isotopic abundances in the Dy fraction, a ^161^Dy/^158^Dy ratio of 1.61 was deduced. Since the amount of ^161^Dy in the Tb fraction corresponds to 1.90 ppb, a ^158^Dy = 1.18 ppb is calculated. The signal at A = 158 Dalton in the Tb fraction corresponds to 24 ppb. Hence, the contribution of ^158^Dy to the ^158^Tb-^158^Dy isobaric interference is less than 5%. Overall, the mass fraction of Dy in the Tb fraction is 1%.

The total amounts of ^146^Sm, ^148,150^Gd, ^154^Dy, ^157,158^Tb contained in the Sm, Gd, Dy, and Tb samples collected with Method B and analyzed by ICP-MS are listed in Table 8, together with the minimum amount of material needed for half-life determination studies.

**Table 8:** Amount (in number of atoms, N_a_) of ^146^Sm, ^148,150^Gd, ^154^Dy, ^157,158^Tb collected in this work by applying Method B. The minimum amount of material (in N_a_) required for performing half-life measurements are indicated as well.

Nuclide	N_a_ retrieved	N_a_ required
^154^Dy	4.07E+15	1.366E+14
^148^Gd	9.64E+14	3.396E+9
^150^Gd	2.38E+15	8.286E+13
^146^Sm	7.06E+15	4.553E+15
^157^Tb	1.42E+15	4.507E+9
^158^Tb	1.75E+14	8.195E+9

The remaining irradiated Ta samples (equivalent overall to 45.29 g of Ta material) will be processed by applying the separation Method B.

## Conclusions

A radiochemical separation method for retrieving ppb level of Sm, Gd, Tb, and Dy from Ta targets irradiated with proton and spallation neutrons was developed. It was proven that the removal of Ba, Hf, and Lu at the beginning of the process was necessary not only to minimize the overall dose rate exposure (in the mSv/h range), but also to obtain pure Dy and Tb fractions (i.e., devoid of Lu and Hf contaminants). ICP-MS analysis showed a disruption of the natural isotopic abundances, with a predominance of neutron-deficient nuclides such as ^146^Sm, ^148,150^Gd, ^154^Dy, ^157,158^Tb. No isobaric interference was determined in the purified Sm, Gd and Dy fractions. In the Tb fraction, 1% of Dy (mass fraction) was detected. However, the contribution of ^158^Dy to the ^158^Tb-^158^Dy isobaric interference was reported as less than 5%. The purified lanthanide fractions are suitable for the preparation of samples for nuclear physics experiments, i.e., for the preparation of α- or γ-sources for half-life measurements. During the separation process, fractions containing Ba, Hf, Lu, Ho, Eu, and Pm radionuclides were collected as well. Further separation of these fractions is envisaged.

## Supporting information

S1 File (ZIP)

## References

[1] Rauscher T. Solution of the α-potential mystery in the γ-process and its impact on the Nd/Sm ratio in meteorites. Physical review letters. 2013 111(6):061104 10.1103/PhysRevLett.111.061104 23971552

[2] Rauscher T. Branchings in the γ-process path revisited. Physical Review C. 2006 73(1):015804.

[3] Mohr P. Photon‐induced Reactions in Stars and in the Laboratory: A Critical Comparison. In AIP Conference Proceedings 2004 (Vol. 704, No. 1, pp. 532–539). American Institute of Physics.

[4] Tanoue Y, Yokoyama T, Ozawa M. Resource evaluation of heavy rare earth derived from the spent Gd2O3 burnable poison in LWRs. Journal of Energy and Power Engineering. 2016 10(4):237.

[5] Filianin PE, Blaum K, Eliseev SA, Gastaldo L, Novikov YN, Shabaev VM, et al On the keV sterile neutrino search in electron capture. Journal of Physics G: Nuclear and Particle Physics. 2014 41(9):095004.

[6] Möller S, Wegener T. Production of nuclear sources and nuclear batteries by proton irradiation. arXiv preprint arXiv:1608.05199. 2016.

[7] Dunlavey DC, Seaborg GT. Alpha activity of Sm-146 as detected with nuclear emulsions. Physical Review. 1953 92(1):206.

[8] Friedman A. M., Milsted J., Harkness A. L. α-Decay Half-Lives of Gd-148, Gd-150, and Sm-146. Bulletin of the American Physical Society. 1963 8(7):525.

[9] Nurmia M., Graeffe G., Valli K., Aaltonen J. Alpha activity of Sm-146. Annales Academiæ Scientiarum Fennicæ, Series A. 1964 148(6):190.

[10] Friedman AM, Milsted J, Metta D, Henderson D, Lerner J, Harkness AL, et al Alpha Decay Half Lives of ^148^Gd, ^150^Gd, and ^146^Sm. Radiochimica Acta. 1966 5(4):192.

[11] Meissner F, Schmidt-Ott WD, Ziegeler L. Half-life and α-ray energy of ^146^Sm. Zeitschrift für Physik A Atomic Nuclei. 1987 327(2):171.

[12] Kinoshita N, Paul M, Kashiv Y, Collon P, Deibel CM, DiGiovine B, et al A shorter ^146^Sm half-life measured and implications for ^146^Sm-^142^Nd chronology in the solar system. Science. 2012 335(6076):1614 10.1126/science.1215510 22461609

[13] Holden NE. Long-lived heavy mass elements half-lives (A> 125). Brookhaven National Lab, Upton, NY (USA); 1985 10.1038/nature03336

[14] Mahunka I, Fenyes T. Investigation of the Alpha Spectrum of Dy Isotopes. Bulletin of the Academy of Sciences of the USSR: Physics Series. 1966 29:1126.

[15] Golovkov N A, Gromov K Y, Lebedev N A, Makhmudov B, Rudnev A S, Chumin V G. Concerning Alpha Decay of Dy, Tb, Cd and Eu Isotopes. Bulletin of the Academy of Sciences of the USSR: Physics Series. 1968 31:1657

[16] Iwata S, Fujiwara I, Nishi T, Goda S, Tabushi M, Shigematsu T. The Half Life of ^157^Tb. Journal of the Physical Society of Japan. 1963 18(2):315.

[17] Grigorev E P. Half Life of Tb-157. Soviet Journal of Experimental and Theoretical Physics. 1964 19:770.

[18] Fujiwara I, Iwata S, Nishi T. Decay of ^157^Tb. Nuclear Physics. 1964 50:346.

[19] Beyer GJ, De Rújula A, Von Dincklage RD, Gustafsson HÅ, Hansen PG, Hoff P, et al The q-value of ^157^Tb. Nuclear Physics A. 1983 408(1):87.

[20] Helmer RG. Nuclear data sheets for A = 157. Nuclear Data Sheets. 1996 78(2):219.

[21] Macfarlane RD. Dysprosium-154, a long-lived α-emitter. Journal of Inorganic and Nuclear Chemistry. 1961 19(1–2):9.

[22] Gono Y, Hiruta K. Non-Existence of Isomeric α-Decay in ^154^Dy. Journal of the Physical Society of Japan. 1971 30(5):1241.

[23] Raman S, Campbell JL, Prindle A, Gunnink R, Palathingal JC. Unsuitability of Tb-157 for light-neutrino rest-mass studies. Physical Review C. 1992 46(6):2241. 10.1103/physrevc.46.2241 9968350

[24] Siivola A. On the alpha activity of neutron deficient europium and gadolinium isotopes. Annales Academiæ Scientiarum Fennicæ, Series A. 1962 190(6).

[25] Ogawa I, Doke T, Miyajima M, Nakamoto A. Alpha decay of Gd-150. Nuclear Physics. 1965 66(1):119.

[26] Lewis HR, Naumann RA, Power JL. Electron Capture Decay of ^158^Tb. Bulletin of the American Physical Society. 1961 6(3):238.

[27] Propsero JM. Studies of Radioactive Decay in the Region of Deformed Nuclei. Thesis, Princeton University. 1963. PUC-1963-95.

[28] Prestwood RJ, Curtis DB, Rokop DJ, Nethaway DR, Smith NL. Determination of the cross section for ^159^Tb(n,2n)^158^Tb and the half-life of ^158^Tb. Physical Review C. 1984 30(3):823.

[29] Zs Fulop, Bartha L Gyurky Gy, Somorjai E, Kubono S, Kudo H, et al The half-life of ^148^Gd. Nuclear Physics A. 2003 718:688.

[30] Prestwood RJ, Curtis DB, Cappis JH. Half-life of Gd-148. Physical Review C. 1981 24(3):1346.

[31] Schumann D, Neuhausen J. Accelerator waste as a source for exotic radionuclides. Journal of Physics G: Nuclear and Particle Physics. 2007 35(1):014046.

[32] Schumann D, Stowasser T, Dressler R, Ayranov M. Possibilities of preparation of exotic radionuclide samples at PSI for scientific investigations. Radiochimica Acta. 2013 101(8):501.

[33] Dai Y, Jia X, Thermer R, Hamaguchi D, Geissmann K, Lehmann E, et al The second SINQ target irradiation program, STIP-II. Journal of nuclear materials. 2005 343(1–3):33.

[34] Kerl W, Becker JS, Dannecker W, Dietze HJ. Application of on-line HPLC-ICP-MS for the determination of the nuclide abundances of lanthanides produced via spallation reactions in an irradiated tantalum target of a spallation neutron source. Fresenius' journal of analytical chemistry. 1998 362(5):433.

[35] Becker JS, Kerl W, Dietze HJ. Nuclide analysis of an irradiated tantalum target of a spallation neutron source using high performance ion chromatography and inductively coupled plasma mass spectrometry. Analytica chimica acta. 1999 387(2):145.

[36] Day JA, Caruso JA, Becker JS, Dietze HJ. Application of capillary electrophoresis interfaced to double focusing sector field ICP-MS for nuclide abundance determination of lanthanides produced via spallation reactions in an irradiated tantalum target. Journal of Analytical Atomic Spectrometry. 2000 15(10):1343.

[37] Nash KL. A review of the basic chemistry and recent developments in trivalent f-elements separations. Solvent Extraction and Ion Exchange. 1993 11(4):729.

[38] Nash KL, Jensen MP. Analytical-scale separations of the lanthanides: A review of techniques and fundamentals. Separation Science and Technology. 2001 36(5–6):1257.

[39] Bhattacharyya A, Mohapatra PK. Separation of trivalent actinides and lanthanides using various ‘N’,‘S’ and mixed ‘N, O’ donor ligands: a review. Radiochimica Acta. 2019 107(9–11):931.

[40] Thomas Κ. Recovery and isolation of Curie quantities of hafnium and the lanthanides from LAMPF-irradiated tantalum targets. Radiochimica Acta. 1983 34(3):135.

[41] Thomas, K., Separation of hafnium and the lanthanides from a tantalum target. In: ollected Radiochemical and Geochemical Procedures Fifth Edition. Kleinberg, J. Editor. United States: N. p., 1990. Web. 10.2172/7003813

[42] Prestwood RJ, Curtis DB, Cappis JH. Half-life of Gd-148. Physical Review C. 1981 24(3):1346.

[43] Talip Z, Dressler R, David JC, Vockenhuber C, Müller Gubler E, Vögele A, et al Radiochemical Determination of Long-Lived Radionuclides in Proton-Irradiated Heavy-Metal Targets: Part I- Tantalum. Analytical chemistry. 2017 89(24):13541 10.1021/acs.analchem.7b03952 29119788

[44] Nucleonica GmbH Nucleonica Nuclear Science Portal (www.nucleonica.com), Version 3.0.65, Karlsruhe (2017).

[45] Müller EI, Mesko MF, Moraes DP, Maria das Graças AK, Flores ÉM. Wet digestion using microwave heating In: Microwave-assisted sample preparation for trace element analysis. 2014 pp. 99–142. Elsevier.

[46] Aksenov NV, Bozhikov GA, Berdonosov SS, Lebedev VY, Dmitriev SN. Coprecipitation of Ti, Zr, and Hf as Rf homologs with La fluoride from solutions of hydrofluoric acid. Physics of Particles and Nuclei Letters. 2011 8(4):356.

[47] Santoyo E, García R, Galicia-Alanis KA, Verma SP, Aparicio A, Santoyo-Castelazo A. Separation and quantification of lanthanides in synthetic standards by capillary electrophoresis: A new experimental evidence of the systematic “odd–even” pattern observed in sensitivities and detection limits. Journal of Chromatography A. 2007 1149(1):12 10.1016/j.chroma.2007.03.036 17400236

[48] McAlister DR, Horwitz EP. Characterization of extraction of chromatographic materials containing Bis(2‐ethyl‐1‐hexyl)Phosphoric Acid, 2‐Ethyl‐1‐Hexyl(2‐Ethyl‐1‐Hexyl)phosphonic acid, and Bis(2, 4, 4‐Trimethyl‐1‐Pentyl)phosphinic acid. Solvent Extraction and Ion Exchange. 2007 25(6):757.

[49] Ekatova TY, Kazakov AG. Extraction-chromatographic behavior of Zr(IV) and Hf(IV) on TRU and LN resins in mixtures of HNO3 and HF. Journal of Radioanalytical and Nuclear Chemistry. 2019 321(2):557.

[50] Horwitz EP, Bloomquist CA. Chemical separations for super-heavy element searches in irradiated uranium targets. Journal of Inorganic and Nuclear Chemistry. 1975 37(2):425.

[51] Pourmand A, Dauphas N. Distribution coefficients of 60 elements on TODGA resin: application to Ca, Lu, Hf, U and Th isotope geochemistry. Talanta. 2010 81(3):741 10.1016/j.talanta.2010.01.008 20298848

